# Programmed Exercise Attenuates Familial Hypertrophic Cardiomyopathy in Transgenic E22K Mice *via* Inhibition of PKC-α/NFAT Pathway

**DOI:** 10.3389/fcvm.2022.808163

**Published:** 2022-02-21

**Authors:** Haiying Wang, Yuedong Lin, Ran Zhang, Yafen Chen, Wei Ji, Shenwei Li, Li Wang, Rubin Tan, Jinxiang Yuan

**Affiliations:** ^1^Department of Physiology, Institute of Basic Medical College, Jining Medical University, Jining, China; ^2^Cardiac Emergency Department, Affiliated Hospital of Jining Medical University, Jining, China; ^3^Institute of Basic Medical College, Jining Medical University, Jining, China; ^4^School of Nursing, Medical College, Soochow University, Suzhou, China; ^5^Department of Physiology, Basic Medical School, Xuzhou Medical University, Xuzhou, China; ^6^The Collaborative Innovation Center, Jining Medical University, Jining, China

**Keywords:** E22K, PKC-α, NFAT, fibrosis, hypotrophy

## Abstract

Familial hypertrophic cardiomyopathy (FHCM), an autosomal dominant disease, is caused by mutations in genes encoding cardiac sarcomeric proteins. E22K, a mutation in the myosin regulatory light chain sarcomere gene, is associated with the development of FHCM. However, the molecular mechanisms by which E22K mutation promotes septal hypertrophy are still elusive. The hypertrophic markers, including beta-myosin heavy chain, atrial natriuretic peptide and B-type natriuretic peptide, were upregulated, as detected by fluorescence quantitative PCR. The gene expression profiles were greatly altered in the left ventricle of E22K mutant mice. Among these genes, nuclear factor of activated T cells (*NFAT*) and protein kinase C-alpha (*PKC-*α) were upregulated, and their protein expression levels were also verified to be elevated. The fibrosis markers, such as phosphorylated Smad and transforming growth factor beta receptor, were also elevated in transgenic E22K mice. After receiving 6 weeks of procedural exercise training, the expression levels of PKC-α and NFAT were reversed in E22K mouse hearts. In addition, the expression levels of several fibrosis-related genes such as transforming growth factor beta receptor 1, Smad4, and alpha smooth muscle actin in E22K mouse hearts were also reversed. Genes that associated with cardiac remodeling such as myocyte enhancer factor 2C, extracellular matrix protein 2 and fibroblast growth factor 12 were reduced after exercising. Taken together, our results indicate that exercise can improve hypertrophy and fibrosis-related indices in transgenic E22K mice *via* PKC-α/NFAT pathway, which provide new insight into the prevention and treatment of familial hypertrophic cardiomyopathy.

## Introduction

Familial hypertrophic cardiomyopathy (FHCM) is an autosomal dominant genetic disease featured by myofibrillar disarray and left ventricle or septal hypertrophy (1, 2). It originates from gene mutations encoding major proteins of myocardial filaments (e.g., myosin) and other related proteins. E22K (glutamic acid to lysine substitution at position 22), a genetic mutation in myosin regulatory light chain (RLC) sarcomeric protein, is located near the Ca^2+^ binding site and RLC phosphorylation site (Ser15) (3). The E22K mutation in RLC gene may cause hypertrophic cardiomyopathy with septal hypertrophy and midventricular obstruction (3), and is closely associated with the eventual formation of FHCM. Previous research has shown that E22K mutation prevents RLC phosphorylation and decreases its affinity for Ca^2+^ binding (3). In transgenic E22K mouse model, the left ventricular systolic and diastolic, ejection or shortening fractions were not affected by E22K mutation, the heart-to-body weight ratio was relatively the same, and the M-mode echo did not show any signs of hypertrophy. However, hematoxylin and eosin staining data showed that 13-month-old E22K mutant mice had the characteristics of enlarged ventricular septa and papillary muscle (2). Other physiological studies indicated that the Ca^2+^ activated force and myofibrillar ATPase activity in E22K mice were increased compared to those in wild-type mice (2).

Protein kinase C (PKC) is a calcium dependent serine/threonine kinase family that can be elicited *via* hydrolysis of membrane phosphatidylinositol and receptor-mediated activation of phospholipase C (PLC) (4). PKC has generally been considered as a master regulator of signal transduction pathway, which regulates various biological functions, including cell growth, differentiation, transformation, apoptosis, tumorigenesis, and so on (5, 6). PKC is a central enzyme that regulates the proliferation and hypertrophy of cardiomyocytes, and plays an essential role in mediating cardiac signal transduction. It has been reported that PKC signaling pathway can be activated by the intracellular events related to circulating hormone response. These events affect different physiological processes of the cardiovascular system, leading to time-varying and force-dependent effects (7). Numerous studies have demonstrated that PKC activation is related to cardiovascular diseases, including heart failure (8–12). The main subtype of PKC expressed in rabbit, mouse and human hearts is PKC-alpha (PKC-α). It has been reported that the activation or overexpression of PKC-α is associated with cardiac hypertrophy, mitogenic stimulation, dilated cardiac myopathy, ischemic injury, myocardial infarction, and human heart failure (13–15). PKC-α triggers the activation of different regulatory proteins, such as the nuclear factor of activated T cells (NFAT) and cyclic adenosine monophosphate (cAMP) response-binding protein (CREB), which are also Ca^2+^ responsive transcription factors (16). The dephosphorylated NFAT can promote the expression of various genes in neurons, cardiac muscle cells, lymphocytes, and skeletal muscle cells (17–19).

Although ventricular hypertrophy caused by E22K mutation has been extensively studied in cell and animal models, the molecular mechanism of myocardial remodeling caused by E22K mutation is still elusive. We hypothesize that the activation of PKC-α/NFAT signaling pathway caused by E22K mutation may lead to cardiomyocyte remodeling. Growing evidence indicates that cardiac rehabilitation is associated with the decreased incidence and mortality of cardiovascular diseases (20, 21). Hypertrophic cardiomyopathy can also be improved by regular exercise, especially aerobic exercise (22). This study aimed to investigate whether the expression levels of genes and proteins related to myocardial remodeling can be reversed in E22K mutant mice after receiving a programmed exercise.

## Materials and Methods

### Mouse Models

Transgenic mice were procured from Dr. Danuta Szczesna-Cordary's laboratory (FL, USA). The transgenic knock-in mice had the expression of myc-tagged human RLCs driven by α-myosin heavy chain promoter. The myc-E22K (Tg-E22K) mutant (2), myc-wild-type (Tg-WT), and non-transgenic (Tg-WT) B6SJL mice were obtained. The animal experiments were approved by the Institutional Animal Care and Use Committee at the Soochow University (reference number: BK20150353), and were performed in compliance with the Guidelines for the Care and Use of Research Animals established by the Soochow University, Suzhou, China. All mice were placed in 12/12 h of dark/bright cycles under specific aseptic (SPF) conditions. They were given unlimited access to water and food throughout the experiment.

### Animal Experiments

The Tg-E22K, Tg-WT, and Non-Tg mice (male, 8 weeks old) were randomly assigned to treadmill exercise group (E-Tg-E22K, E-Tg-WT, and E-Non-Tg) and rest (sedentary) control group (R-Tg-E22K, R-Tg-WT, and R-Non-Tg). Mice in the exercise group were introduced to running exercise (4 days per week on a treadmill) for 6 weeks at 20°C. Adaptive training was performed with moderate treadmill running (first ran at 5 m/min for 5 min, and then ran at 10 m/min for another 5 min) 1 day before initiating the exercise program. In the 6-week exercise training, warming-up and cooling-down (5 min running at 5 m/min) were conducted, followed by the running exercise program of 10 min at 10 m/min at week 1, 10 min at 15 m/min week 2, 20 min at 15 m/min at week 3 and 4, and 20 min at 20 m/min at week 5 and 6. Mice in the rest group were placed on the stationary treadmill for 5–10 min for 4 times per week (23). All mice were euthanized by cervical dislocation 3 days after exercise. The heart tissues were collected immediately, and then placed into cold phosphate buffer saline solution to remove any residual blood. All tissue samples were stored in a freezer at −80°C until further analysis.

### RNA Sequencing

To reveal the transcriptome changes induced by E22K mutation, TRIzol reagent (Thermo Fisher Scientific, USA) was used to extract total RNA from the heart tissues of mice with different genotypes, including Tg-WT, Tg-E22K, and Non-Tg. The library construction and RNA sequencing were conducted by Novogene Company (Beijing, China).

### cDNA Synthesis and Real-Time PCR

Total RNA was extracted from the left ventricular myocardial tissues of three genotypes (Tg-E22K, Tg-WT, and Non-Tg) using Trizol reagent (Thermo Fisher Scientific, Catalog #15596026). After treatment with DNASE-I (New England Biolabs, Catalog #M0303S) to remove genomic DNA, total RNA was cleaned with RNA clean-up Kit (Qiagen, Catalog #74204). cDNA synthesis was conducted with PrimeScript TM RT Master Mix (RR036A, Tkara, China) reaction system according to the manufacturer's instructions. Real-time PCR amplification was conducted by TB Green Premix Ex TaqTM II (RR820A, Tkara, China) reaction system using the primers listed in Table 1 (24). The 2^−Δ*ΔCt*^ method was used for the relative quantification of gene expression, standardized by glyceraldehyde-3-phosphate dehydrogenase (GAPDH) as the invariant control for the sample, and then compared with the reference sample.

**Table 1:** The primer pairs used for real-time PCR.

**Name**	**q-PCR primer**
mGAPDH	F: 5′-CATTTCCTGGTATGACAATGAATACG-3′
	R: 5′-TCCAGGGTTTCTTACTCCTTGGA-3′
β-MHC	F: 5′-TGCAAAGGCTCCAGGTCTGAGGGC-3′
	R: 5′-GCCAACACCAACCTGTCCAAGTTC−3′
ANP	F: 5′-AGGAGAAGATGCCGGTAGAAGA-3′
	R: 5′-GCTTCCTCAGTCTGCTCACTCA-3′
BNP	F: 5′-GTCTTGGCCTTTTGGCTTC-3′
	R: 5′-TTCCTCAGTCTGCTCACTC-3′

### Western Blotting

Total protein of the left ventricular myocardium tissues from three genotype of mice (Tg-E22K, Tg-WT, and Non-Tg) was lysed in 1 × RIPA buffer (Bio-Rad). Protein quantification was performed using the BCA protein assay reagent according to manufacturer's instruction. Protein extracts (25 μg/lane) were separated by SDS-PAGE using 10% lab-made Tris-glycine gels, and transferred onto a PVDF membrane. The membranes were blocked with 5% dried milk for 1 h at room temperature, followed by overnight incubation with primary antibody. The primary antibodies were diluted with 1X TBS+Tween (TBST) containing 3% bovine serum albumin (BSA). After washing three times with TBST buffer (10 min/each time), the membranes were incubated with the corresponding secondary antibody in TBST containing 5% dried milk at room temperature for 1 h. Visualization of protein blots was performed using the ECL substrate (Tanon 5200) after three times washing with TBST (10 min/each time). The primary antibodies used were as follows: GAPDH (D16H11, 1:1000, 5174S, Cell Signaling Technology, USA), PKC-α (D7E6E, 1:1000, 59754S, Cell Signaling Technology, USA), NFAT1 (D43B1, 1:1000, 5861S, Cell Signaling Technology, USA), Smad2/3 (D7G7, 1:1000, 8685s, Cell Signaling Technology, USA), Phospho-SMAD2 (Ser465/Ser467, 1:1000, 8685s, Cell Signaling Technology, USA), TGFβ Receptor II (1:1000, 79424s, Cell Signaling Technology, USA). The secondary antibodies used were HRP-linked goat anti-Mouse IgG (1:5000, ZB-2305, ZSGB-Bio, China) and Goat Anti-rabbit IgG (1:5000, ZB-2305, ZSGB-Bio, China). Densitometry analysis was conducted with ImageJ 1.34s software.

### Statistical Analysis

Data are presented as mean ± SEM. Unpaired, two-sided Student's *t*-test was used when comparing 2 sets of data as indicated. Meanwhile, unpaired, one-way ANOVA followed by Tukey's *post-hoc* test was used for multiple comparisons. All statistical tests were performed using the GraphPad Prism8 software. *P* < 0.05 was defined as statistical significant.

## Result

### E22K Mutation Induces Ventricular Hypertrophy

**Figure 1:**
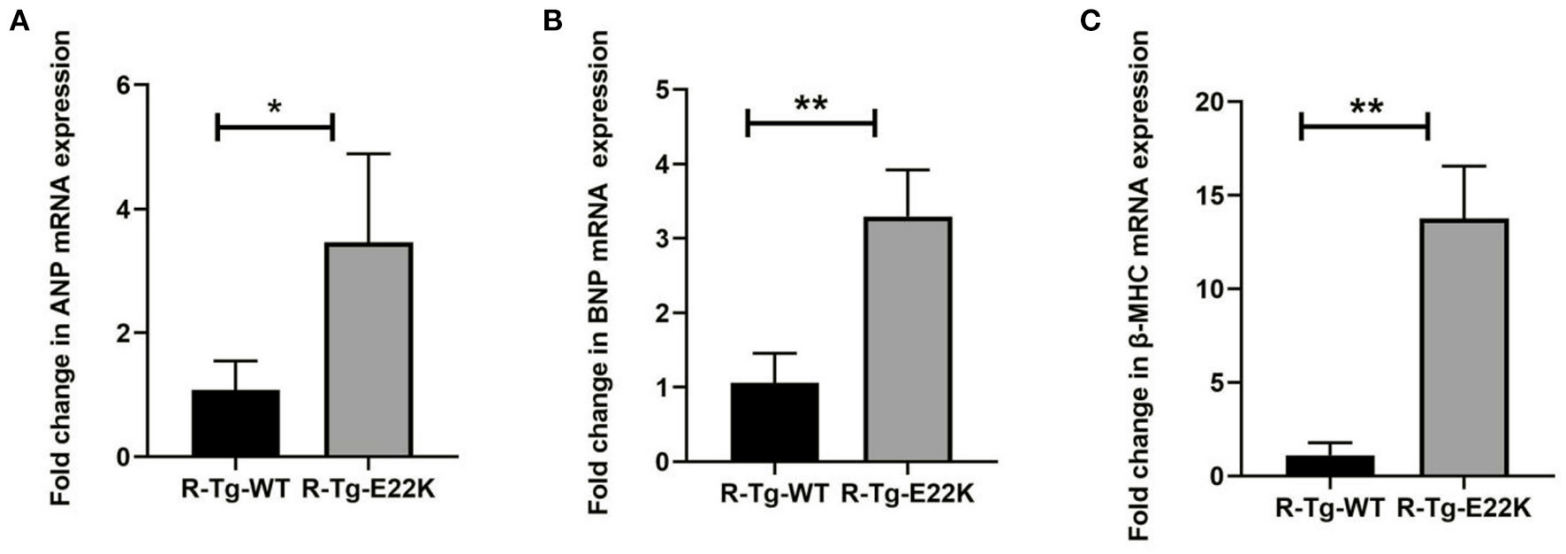
E22K mutation significantly increases the expression levels of ventricular hypertrophy markers. **(A–C)** Real-time PCR analysis was conducted for the R-Tg-E22K and R-Tg-WT mice. The mRNA levels of ANP **(A)**, BNP **(B)**, and β-MHC **(C)** were determined using the delta-delta CT method. Compared to R-Tg-WT mice, the expression levels of these three genes were remarkably upregulated in R-Tg-E22K mice heart tissue. Each sample was repeated three times. Student's *t*-test was employed to assess the differences in abundance levels. *N* = 3, ^*^p < 0.05; ^**^p < 0.01. R-Tg-WT, wild-type transgenic mice in the rest group; R-Tg-E22K, transgenic E22K mice in the rest group.

To determine the effect of E22K mutation on ventricular hypertrophy, real-time PCR was performed to detect three classic hypertrophic markers in the cardiac tissues of sedentary mice. The hypertrophic markers detected are beta-myosin heavy chain (β-MHC), atrial natriuretic peptide (ANP) and brain natriuretic peptide (BNP). ANP plays an important biological role in inhibiting myocardial hypertrophy and fibrosis, resisting oxidative stress, as well as preventing the development of ischemia/reperfusion injury (25–27). BNP levels are closely related to the degree of left ventricular failure in patients with heart failure (28, 29). β-MHC molecules are important parts of myosin, which play a major role in myocardial contraction (30, 31). It was found that the mRNA levels of β-MHC, ANP, and BNP were upregulated in the cardiac tissues of R-Tg-E22K mice compared to R-Tg-WT mice, indicating that E22K mutation could activate cardiac remodeling (Figures 1A–C).

### E22K Mutation Affects the Pathological Makers of Cardiac Fibrosis

**Figure 2:**
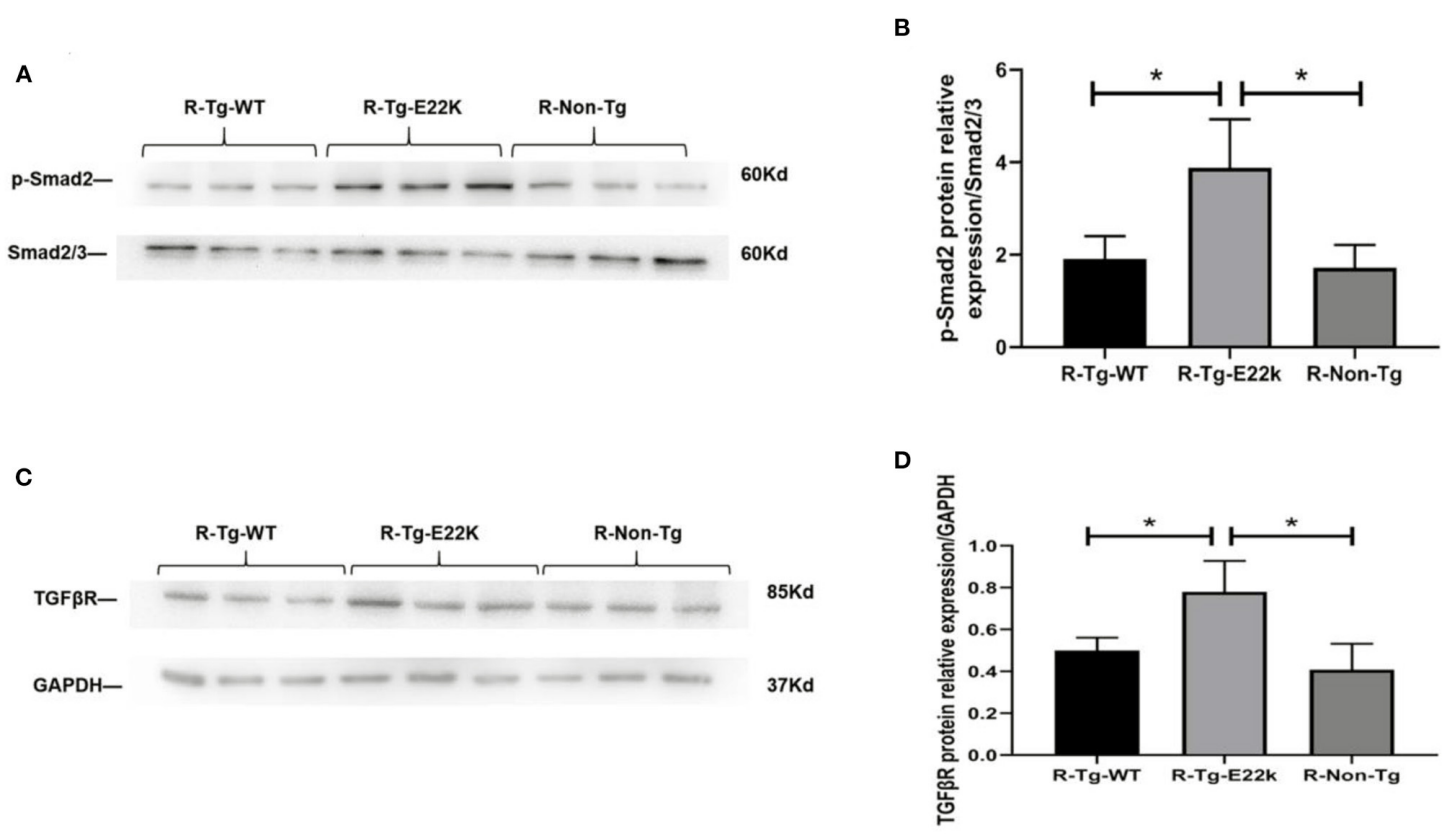
E22K mutation elevates the protein expression of p-Smad2 and TGFβR. Representative Western blot results demonstrated the upregulated expression of TGFβR and increased phosphorylation of Smad2 in R-Tg-E22K mouse hearts compared to those in R-Tg-WT and Non-Tg mouse hearts **(A,C)**. Densitometric analysis of the protein levels of p-Smad2 and TGFβR in each group **(B,D)**. *N* = 3, ^*^p < 0.05. R-Tg-WT, wild-type transgenic mice in the rest group; R-Tg-E22K, transgenic E22K mice in the rest group; R-Non-Tg, non-transgenic wild-type mice in the rest group.

Fibrosis is often associated with ventricular hypertrophy and impairment of cardiac function. As presented in Table 2, fibrosis-related genes such as transforming growth factor beta receptor (*TGF*β*R*), *Smad*, and *Col1a* were significantly upregulated in E22K mice. TGFβ expression in cardiomyocytes may also responsible for cardiac dysfunction and maladaptive hypertrophy *via* regulation of PKC (32, 33). In cardiac fibroblasts, TGFβ plays a central role in regulating different aspects of fibrosis such as myofibroblast differentiation, inflammatory response, extracellular matrix synthesis and gene expression (34–36). TGFβ receptor transmits intracellular signals through Smad proteins, which in turn translocate to the nucleus to control gene transcription (37). The expression levels of TGFβR and p-Smad2 proteins were verified by Western blotting to determine their association with fibrosis in E22K transgenic mouse hearts. Western blot results showed that Smad2 phosphorylation and TGFβR levels were upregulated in R-Tg-E22K mouse heart tissues compared to R-Tg-WT and Non-Tg control mouse heart tissues (Figures 2A,C). Quantitative analysis indicated there were significant differences in the protein levels of p-Smad2 and TGFβR (*p* < 0.05, Figures 2B,D). Our results indicated that E22K mutation affected the expression of cardiac fibrosis makers in E22K mice heart.

**Table 2:** Changes in gene expression profiles in the left ventricle of E22K mutant mice.

**Gene**	**Log2FC**	***P*** **-value**	**P-adj**	**Gene_description**
**R-E22K vs. R-WT**				
Myh6	−0.760203209	8.73E-14	7.48E-11	Myosin, heavy polypeptide 6, cardiac muscle, alpha
Myh7b	−1.089644008	7.79E-19	1.09E-15	Myosin, heavy chain 7B, cardiac muscle, beta
Mhrt	1.715486122	1.52E-26	5.88E-23	Myosin heavy chain associated RNA transcript
Nppa	2.930583503	0.002454211	0.034356725	Natriuretic peptide type A
Nppb	1.678670308	0.005711738	0.062158281	Natriuretic peptide type B
Col6a5	3.375981869	0.00000577	0.000367596	Collagen, type VI, alpha 5
Col8a1	1.277527091	0.0000159	0.000791111	Collagen, type VIII, alpha 1
Col1a2	0.993288854	0.00086216	0.016063451	Collagen, type I, alpha 2
Tgfbr1	0.594576373	0.002391314	0.033782781	Transforming growth factor, beta receptor I
Tgfb2	1.50076272	1.57E-08	0.00000303	Transforming growth factor, beta 2
Tgfb3	1.186157811	0.000000077	0.000012	Transforming growth factor, beta 3
Smad4	0.329231545	0.001305242	0.021839444	SMAD family member 4
Acta2	1.333706795	0.000000816	0.0000801	alpha smooth muscle actin
Prkca	0.38901659	0.002945265	0.039203284	Protein kinase C, alpha
Nfatc4	0.739779165	0.003460926	0.043890785	Nuclear factor of activated T cells, cytoplasmic, calcineurin dependent 4

### E22K Mutation Remarkably Changes Gene Expression Profiles in the Left Ventricle of Mouse Hearts

**Figure 3:**
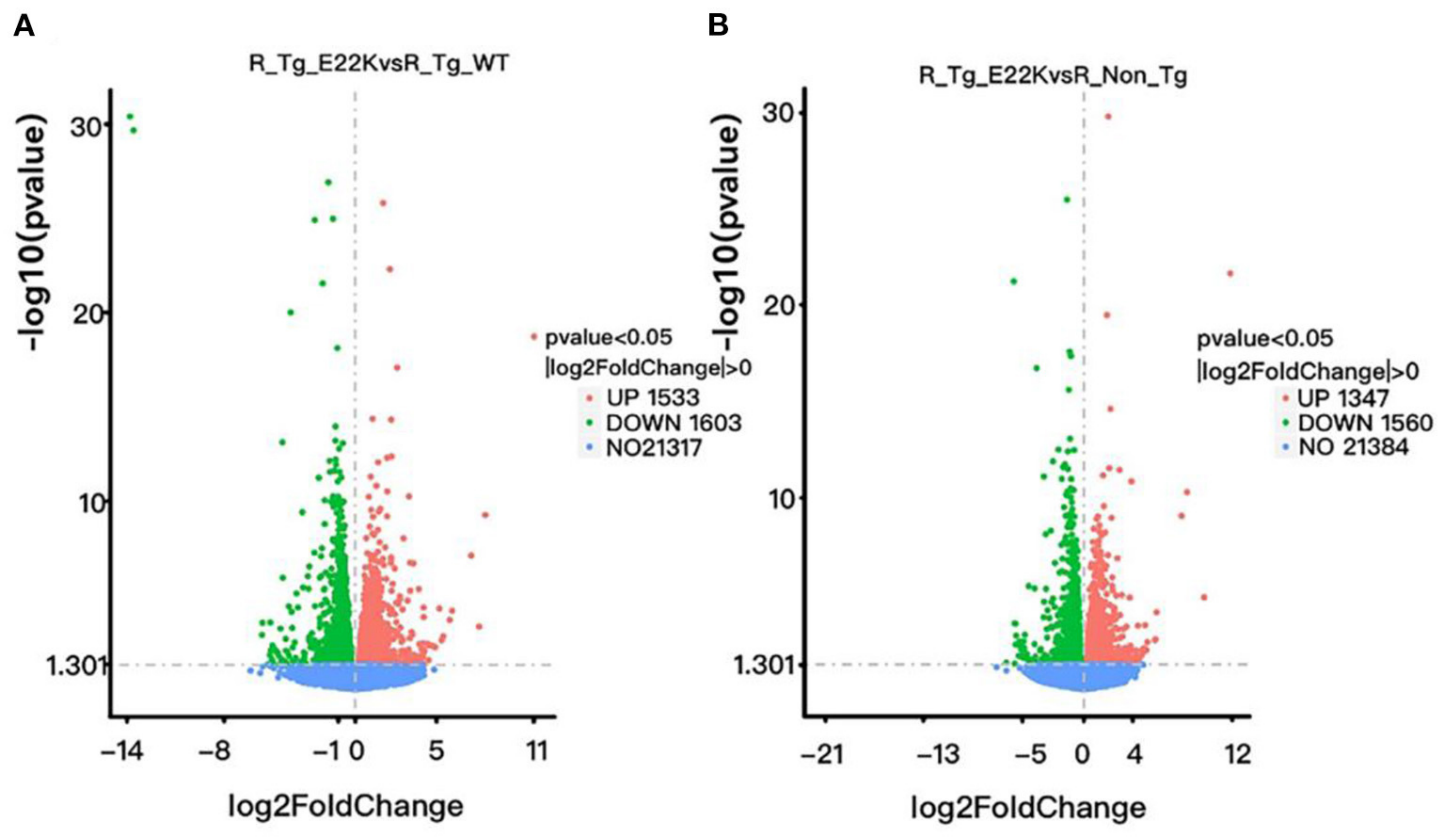
E22K site mutation remarkably changes the gene expression profiles of cardiomyocytes in the left ventricle. Volcano map of DEGs **(A,B)**. There were 3,136 genes differently expressed between R-Tg-E22K and R-Tg-WT groups **(A)**, of which 1,533 and 1,603 genes were upregulated and downregulated, respectively, and 21,317 genes remained unchanged. There were 2,907 genes differently expressed between R-Tg-E22K and R-Non-Tg groups **(B)**, of which 1,347 and 1,560 genes were downregulated and upregulated, respectively, and 21,384 genes were not altered. R-Tg-WT, wild-type transgenic mice in the rest group; R-Tg-E22K, transgenic E22K mice in the rest group; R-Non-Tg, non-transgenic wild-type mice in the rest group.

To investigate whether RLC site mutation at E22K can affect the gene expression profiles of cardiomyocytes in the left ventricle, RNA sequencing was performed after extraction of total RNA from the heart tissues of mice with three different genotypes in the rest group. In total, 3,136 differentially expressed genes (DEGs) were detected between R-Tg-E22K and R-Tg-WT mice, of which 1,533 were upregulated and 1,603 were downregulated (Figure 3A). In addition, 21,317 genes remained unchanged. Similar results were noticed between R-Tg-E22K and R-Non-Tg mice. In this group, 2,907 genes were differentially expressed, of which 1,347 and 1,560 genes were upregulated and downregulated, respectively, and 21,384 genes were not altered (Figure 3B). A previous study reported different phenotypes of septal hypertrophy in the hearts of 13-month-old E22K mice *via* histological staining (2). Although echocardiography did not reveal the changes in ejection fraction and shortening fraction between E22K and WT mice (38), our RNA-Seq data indicated three main categories of genes were differentially expressed in the E22K mice heart (Table 2). They are myocardial contraction and hypertrophy-related genes (e.g., *Myh6, Myh7b, Mhrt, Nppa*, and *Nppb*), cardiac fibrosis genes (e.g., *Col6a5, Col8a1, Col1a2, Tgfbr1, Tgfb2, Tgfb3, Smad4, and Acta2*) and signaling pathways-associated genes (e.g., *Prkca* and *Nfatc4)*.

### E22K Mutation Activates PKC-α/NFAT Signaling

**Figure 4:**
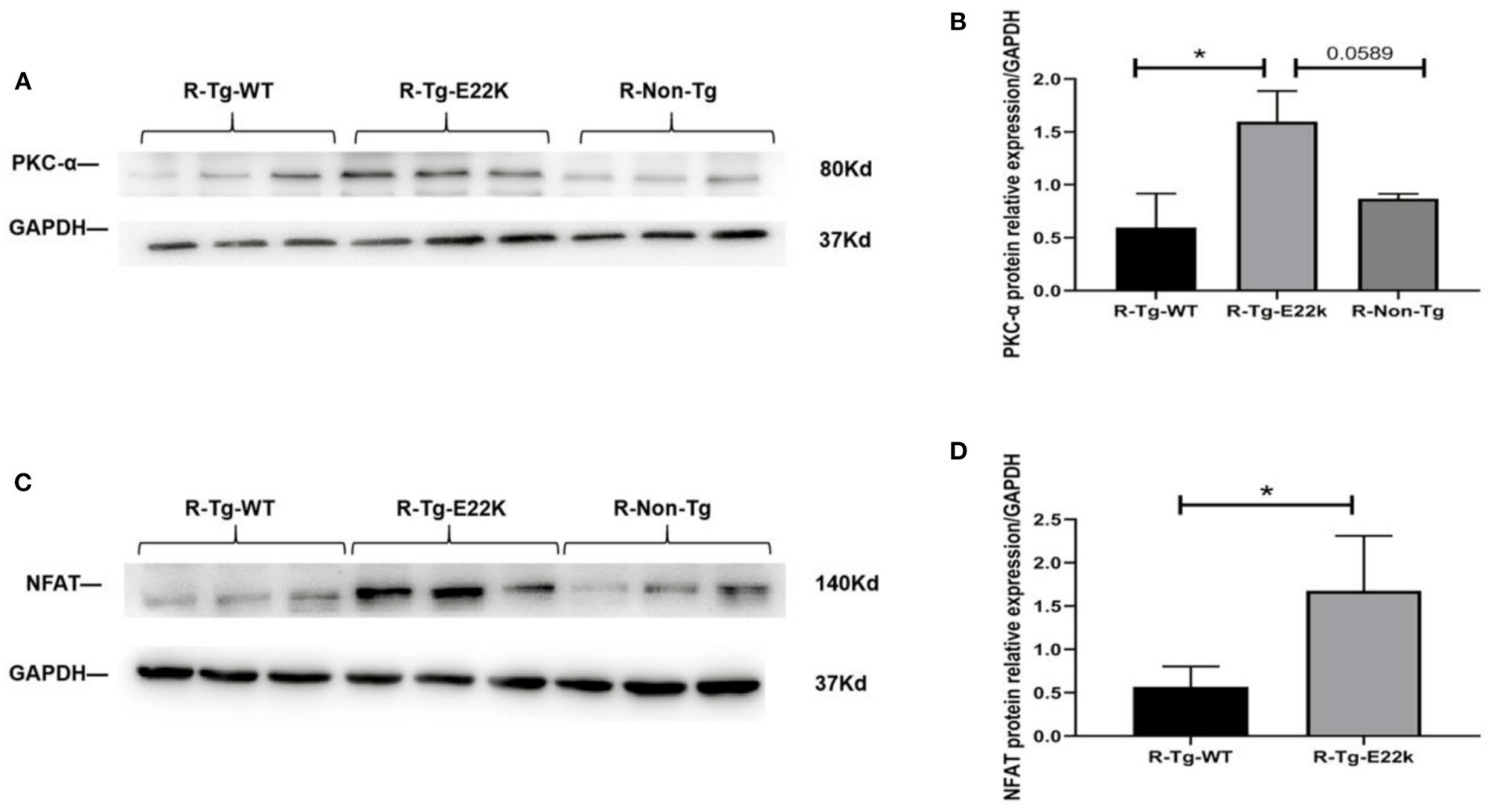
E22K mutation upregulates the protein expression of PKC-α and NFAT. Representative Western blot data showing the upregulated expression levels of PKC-α and NFAT in R-Tg-E22K mouse hearts compared to R-Tg-WT and R-Non-Tg mouse hearts **(A,C)**. Densitometric analysis of the protein levels of PKC-α and NFAT in each group **(B,D)**. *N* = 3, ^*^p < 0.05. R-Tg-WT, wild-type transgenic mice in the rest group; R-Tg-E22K, transgenic E22K mice in the rest group; R-Non-Tg, non-transgenic wild-type mice in the rest group.

As shown in Table 2, PKC-α and NFAT were transcriptionally upregulated in R-Tg-E22K mouse hearts compared to those in R-Tg-WT and Non-Tg mouse hearts. Therefore, Western blotting was carried out to detect the protein levels of PKC-α and NFAT. Our results confirmed that, in transgenic E22K mouse heart tissue, the protein expression levels of PKC-α and NFAT transcription factor were significantly enhanced (*p* < 0.05, Figures 4A–D).

### Exercise Reverses the Protein Expression of PKC-α/NFAT in E22K Mouse Hearts

**Figure 5:**
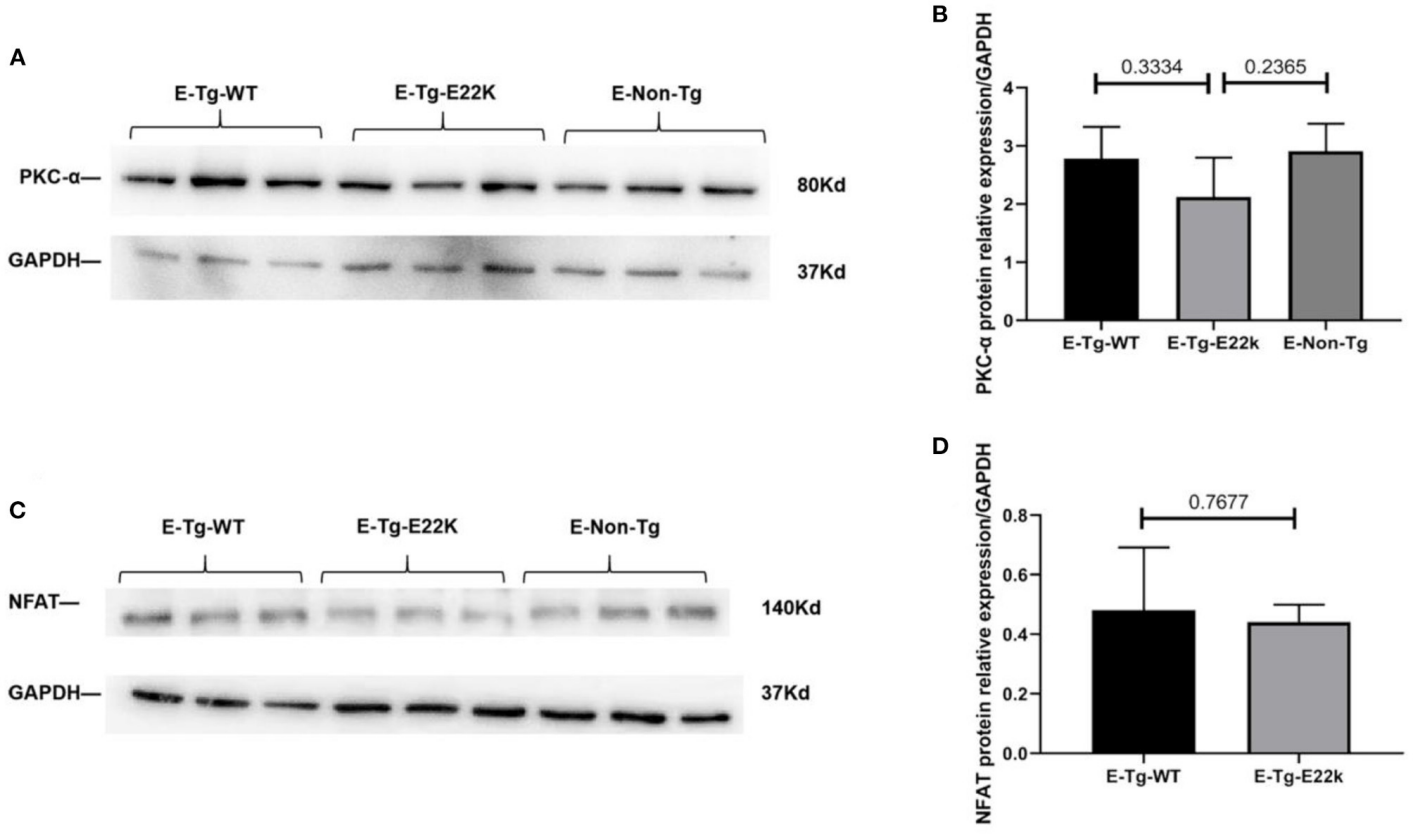
Exercise changes the protein expression of PKC-α/NFAT. Representative Western blot results show that there are no remarkable differences in the expression levels of PKC-α and NFAT between Tg-WT and exercise-trained E22K mice **(A,C)**. Densitometric analysis of the protein levels of PKC-α and NFAT in each group **(B,D)**. *N* = 3, ^*^p < 0.05. E-Tg-WT, wild-type transgenic mice in the exercise group; E-Tg-E22K, transgenic E22K mice in the exercise group; E-Non-Tg, non-transgenic wild-type in the exercise group.

To test whether the expression of PKC-α/NFAT in E22K mouse hearts can be reversed after receiving an exercise training program, Western blot analysis was carried out. Our results showed that the protein levels of PKC-α (Figures 5A,B) and NFAT transcription factor (Figures 5C,D) in exercise-trained E22K mice were relatively similar to those in E-Tg-WT and E-Non-Tg control mice. These results imply that exercise can improve PKC-α/NFAT signaling that is dysregulated due to E22K mutation.

### Exercise Regulates Cardiac Remodeling Related Genes in the Left Ventricle of Both E22K and WT Mice

**Figure 6:**
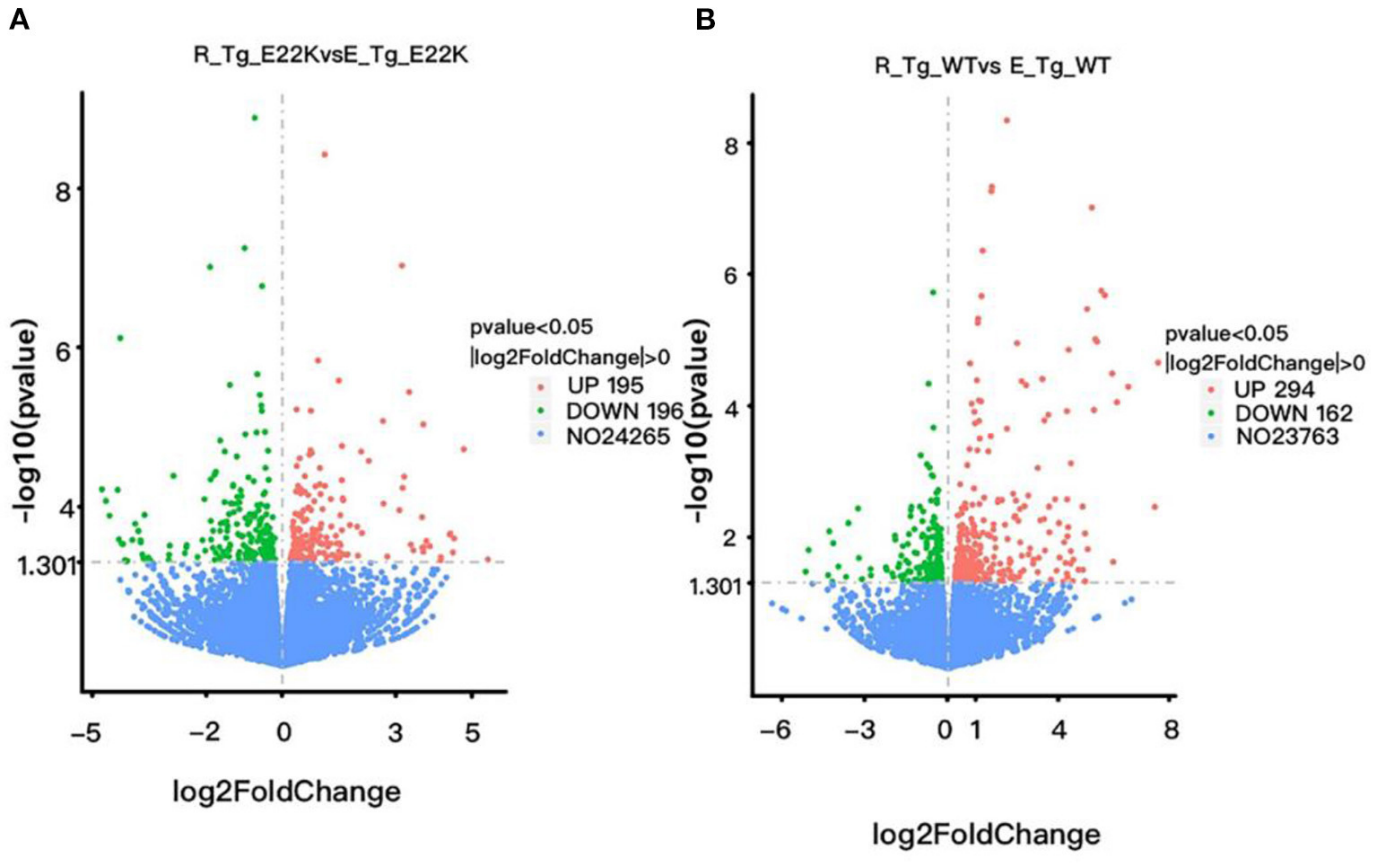
Exercise regulates cardiac remodeling-related genes in mouse hearts. There were 391 genes with differential expression between R-Tg-E22K and E-Tg-E22K groups, of which 195 and 196 genes were upregulated and downregulated, respectively **(A)**. There were 456 genes with differential expression between R-Tg-WT and E-Tg-WT groups, of which 294 and 162 genes were upregulated and downregulated, respectively **(B)**. R-Tg-WT, wild-type transgenic mice in the rest group; R-Tg-E22K, transgenic E22K mice in the rest group; E-Tg-WT, wild-type transgenic mice in the exercise group; E-Tg-E22K, transgenic E22K mice in the exercise group.

To investigate whether exercise can regulate the expression of myocardial function-related genes, all mice were subjected to an exercise training program for 6 weeks. The mouse hearts were then dissected 3 days after performing the exercise program. RNA sequencing was performed on the left ventricle of both the exercise and sedentary mice. Our results indicated that there were 391 genes with differential expression between R-Tg-E22K and E-Tg-E22K groups, of which 195 and 196 genes were upregulated and downregulated, respectively (Figure 6A). Among these genes, the fibrosis-related genes such as fibroblast growth factor 12 (*Fgf12*, Log2FC = −3.71, *p* = 0.0009) and collagen-like tail subunit (*colq*, Log2FC = −0.56, *p* = 0.046), as well as the hypertrophy-related genes such as myocyte enhancer factor 2C (*Mef2*, Log2FC = −0.54, *p* = 0.021), myosin, heavy polypeptide 11 (*Myh11*, LogFC = −0.46, *p* = 0.019), extracellular matrix protein 2 (*Ecm2*, Log2FC = −0.41, *p* = 0.005), cAMP responsive element binding protein 1 (*Creb1*, Log2FC = −0.26, *p* = 0.049), and trans-acting transcription factor 1 (*Sp1*, Log2FC = −0.25, *p* = 0.045) were all downregulated in E22K mice after completing the exercise program (Table 3). These results indicated that exercise could reverse the expression patterns of some fibrosis- and hypertrophy-related genes in E22K mutant mice. In addition, there were 456 genes with differential expression between R-Tg-WT and E-Tg-WT groups, of which 294 and 162 genes were upregulated and downregulated, respectively (Figure 6B). These data further confirm that exercise can regulate the genes related to cardiac remodeling.

**Table 3:** A list of significant DEGs in E22K mice before and after exercise.

**Gene**	**Log2FC**	***P*** **-value**	**Padj**	**Gene_description**
**E-E22K vs. R-E22K**				
Fgf12	−3.711375467	0.000922121	0.999920873	Fibroblast growth factor 12
Colq	−0.558992277	0.045971381	0.999920873	Collagen-like tail subunit
Mef2c	−0.308788186	0.021381395	0.999920873	Myocyte enhancer factor 2C
Myh11	−0.456183295	0.019429391	0.999920873	Myosin, heavy polypeptide 11
Ecm2	−0.412531367	0.00549835	0.999920873	Extracellular matrix protein 2
Creb1	−0.258429962	0.049367256	0.999920873	cAMP responsive element binding protein 1
Sp1	−0.247143887	0.044881631	0.999920873	Trans-acting transcription factor 1

RNA sequencing data indicated that the expression levels of PKC-α and NFAT in E22K mouse hearts did not differ significantly with those in WT mouse hearts after receiving exercise training. This result demonstrates that the dysregulated expression of PKC-α and NFAT in E22K mice is ameliorated after exercise, which is consistent with our Western blot results. In addition, exercise reversed the gene expression of *Tgfbr1, Smad4*, and *Acta2*, which were abnormally elevated in sedentary E22K mice heart (Table 2 vs. Table 4). However, although PKC-α/NFAT and other related genes were remodeled in the hearts of E22K mice, exercise did not modify all hypertrophy-related genes to the same extent as those in WT mouse hearts. The comparative analysis of DEGs in E-Tg-E22K and E-Tg-WT mice showed that most of the hypertrophy- and fibrosis-related genes did not alter following exercise (Table 4). These findings suggest that exercise can partially alleviate familial hypertrophic cardiomyopathy in E22K mutant mice.

**Table 4:** A list of significant DEGs between E22K and WT mice after exercise.

**Gene**	**Log2FC**	***P*** **-value**	**Padj**	**Gene_description**
**E-E22K vs. E-WT**				
Myh7b	−0.737876799	3.76E-06	0.000236149	Myosin, heavy chain 7B, cardiac muscle, beta
Nppa	2.908878667	0.00023003	0.005988536	Natriuretic peptide type A
Nppb	1.353917115	3.37E-08	4.77E-06	Natriuretic peptide type B
Col1a2	1.066315743	2.05E-06	0.000148649	Collagen, type I, alpha 2
Col6a3	0.83315607	1.07E-05	0.000551857	Collagen, type VI, alpha 3
Tgfbr2	0.573306079	0.001080711	0.018510723	Transforming growth factor, beta receptor 2
Tgfb3	1.009221517	3.84E-05	0.001512102	Transforming growth factor, beta 3
Tgfb2	1.784450639	7.85E-11	2.42E-08	Transforming growth factor, beta 2
Smad6	0.532913706	0.001099949	0.018701553	SMAD family member 6
Smad9	−0.679589712	0.022110941	0.141437131	SMAD family member 9

## Discussion

The substitution of glutamic acid to lysine at position 22 (E22K) in RLC gene may cause familial hypertrophic cardiomyopathy (39). A13T, E22K, and P95A are three different RLC mutations that are related to a specific subtype of cardiac hypertrophy characterized by mid-left ventricular obstruction (40). Histological staining revealed the enlarged interventricular and septal papillary muscles in the heart of E22K mutant mice (39).

NFAT and other transcription factors, such as CREB1, are the downstream targets of PKC. Evidence shows that pressure overload-triggered cardiac remodeling is positively correlated with the sustained activation of NFAT, thus leading to heart failure. Myocardial hypertrophy and fibrosis are common symptoms in patients with chronic kidney disease (41). TGFβ is a crucial modulator of fibrosis, which mediates tissue fibrosis by activating its downstream Smad pathway (42). TGFβ/Smad molecular pathway is involved in the pathogenic differentiation from cardiac fibroblasts to myofibroblasts (43). The activation of both TGFβ/Smad and fibroblast growth factor receptor 4 (FGFR4)/PLC gamma (PLC-γ) signaling cascades can result in the development of fibrosis (42, 44–46). PKC-α is one of the most abundantly PKC subtypes in the myocardium. Growing evidence indicates that the overexpression or activation of PKC-α in transgenic mice decreases Ca ^2+^ transients as well as cardiac contractility, which leads to cardiac hypertrophy and ultimately heart failure (47–50). PKC-interacting protein is involved in hypertrophic tissue remolding *via* regulation of calcineurin-NFAT signaling (51). Our study showed that E22K mutation resulted in the upregulated gene expression of hypertrophy and fibrosis markers, and the gene and protein levels of PKC-α and NFAT were significantly elevated in E22K mouse myocardium. In addition, cardiac fibrosis was promoted in E22K mutant mice, as revealed by the increased mRNA and protein expression of fibrosis markers such as p-Smad and TGFβR. Therefore, we speculate that hypertrophy and fibrosis in E22K mouse myocardium may be mediated by PKC-α/NFAT pathway.

Smooth muscle cells (SMC) are one of the important cell types that constitute to the structural and physiological properties of the heart. Vascular smooth muscle cells (VSMC) participate in tissue repair through proliferation, migration and phenotype alteration in response to vascular injury (52). FGF12 has been reported to be a key regulator of VSMC phenotype switch (53, 54). In addition, FGFs have been reported to induce cardiac hypertrophy by activating calcineurin/NFAT signaling (55, 56). Our study indicated that *Fgf12* was downregulated in exercise-trained E22K mice compared to rest E22K mice (Table 3). Downregulation of *Fgf12* may be related to the improvement of cardiac function. Myocyte enhancer factor 2C (Mef2c) is one of the major cardiac regulators that control both muscle and cardiovascular development (57, 58). It has been reported that the enhanced transcriptional activity of NFAT and MEF2 is associated with pathological myocardial hypertrophy (59, 60). A previous study on HCM mouse models demonstrated that exercise could reduce NFAT, but not MEF2 (22). Our data indicated that *Mef2c* was reduced in E22K mice after completing the exercise program (Table 3). This may be due to the different Mef isoforms detected, which function differently. As a highly dynamic non-cellular three-dimensional network, extracellular matrix (ECM) plays crucial roles in modulating cardiac structural homeostasis and cardiac remodeling (61). The abnormal changes in ECM and the structural reconstruction of matrix macromolecules are involved in the pathological process of ventricular hypertrophy, and even heart failure (61). Our data indicated that the expression of *Ecm2* was decreased in E22K mice after performing exercise, indicating a beneficial effect of exercise on cardiac hypertrophy (Table 3).

Exercise is a common rehabilitation training method for individuals with muscle atrophy. Exercise can enhance the function of skeletal muscles. However, the gene expression profiles and signaling mechanisms induced by exercise, especially in patients with hypertrophic heart disease, are still elusive. Cardiac hypertrophy is generally an adaptive response to pathological and physiological stimuli. Pathological hypertrophy often develops into myocardium fibrosis, which eventually leads to heart failure. Previous research has shown that cell development, metabolism, non-coding RNA, immune response, translational activation and epigenetic modification can either positively or negatively regulate myocardial hypertrophy. For patients with pathologically hypertrophic heart disease, the mechanisms underlying the positive effect of exercise on cardiac function remain largely unclear. To explore whether physical exercise has a beneficial effect on cardiac physiology at the molecular level in the E22K mice model, a programmed exercise was scheduled for the mice to verify whether exercise can reverse the characteristics of cardiac hypertrophy. Our results showed that the expression levels of PKC-α and NFAT were relatively similar among E-Tg-E22K, E-Tg-WT, and E-Non-Tg mice after receiving the programmed exercise. It has been reported that exercise is beneficial to hypertrophic cardiomyopathy mice harboring MyHC mutations. NFAT is a marker of myocardial hypertrophy (62). Exercise can prevent the activation of NFAT, and even reverse the established heart disease phenotype (22). Our results are consistent with the previous findings. In addition, our study demonstrated that exercise downregulated the cardiac remodeling-related genes (*fgf12, colq, Mef2C, Ecm2, and Creb1*), and reversed the fibrosis-associated genes (*Tgfbr1, Smad4, and Acta2*), indicating that exercise can improve pathological cardiac remodeling in E22K mutant mice.

## Conclusion

In this study, we elucidated that the elevated expression of PKC-α and NFAT in E22K mouse myocardium may be a potential mechanism of E22K-induced hypertrophy. Furthermore, we verified that a programmed exercise could downregulate cardiac remodeling-related genes (*fgf12, colq, Mef2C, Ecm2*, and *Creb1*), reverse cardiac fibrosis associated genes (*Tgfbr1, Smad4*, and *Acta2*) and inhibit PKC-α/NFAT signaling pathway in the hearts of E22K mutant mice.

### Importance and Significance

In this study, the differences in gene expression profiles between E22K mutant and control mice were compared by RNA sequencing, and the molecular mechanisms of hypertrophy and fibrosis were analyzed. Exercise therapy plays a beneficial role in the improvement of cardiac function. Our study indicated that exercise could decrease the expression levels of *fgf12, colq, Mef2C, Ecm2*, and *Creb1*, all of which were related to cardiac remodeling. In addition, cardiac fibrosis-associated genes, such as *Tgfbr1, Smad4*, and *Acta2*, could be reversed by exercise. Furthermore, our results demonstrated that programmed exercise could reverse the upregulated expression of PKC-α and NFAT caused by E22K mutation in E22K mutant mice at both gene and protein levels. Our study highlights the plasticity of PKC-α and NFAT in mouse hearts during pathological hypertrophy. Most importantly, our study may provide clues to novel strategies into the prevention and treatment of familial hypertrophic cardiomyopathy.

## Data Availability Statement

The datasets presented in this study can be found in online repositories. The names of the repository/repositories and accession number(s) can be found at: SRA, PRJNA786059.

## Ethics Statement

The animal study was reviewed and approved by Institutional Animal Ethics Guidelines for the Care and Use of Research Animals established by Soochow University, Suzhou, China.

## Author Contributions

JY, RT, and LW: conceptualization, experimental design, original draft preparation, and data analysis. HW and YL: perform experiments, data collection and analysis, and review and editing. RZ, YC, WJ, and SL: perform of experiments, data analysis, and editing. All authors have read and approved the final version of the manuscript.

## Funding

The authors would like to acknowledge the Research Start-up Fund of Jining Medical University (Reference: 600791001, JY), the Natural Science Foundation of Jiangsu Province of China (Grant number: BK20150353, LW), the Foundation of Medical Health Science and Technology Development Program of Shandong Province (Reference: 202002061311, HW), the Research Support Fund for Teachers of Jining Medical University (Reference: JYFC2019KJ013, HW), and the Jining Medical College Student Innovation and Entrepreneurship Training Program Project (Reference: cx2021053, HW) for funding this project.

## Conflict of Interest

The authors declare that the research was conducted in the absence of any commercial or financial relationships that could be construed as a potential conflict of interest.

## Publisher's Note

All claims expressed in this article are solely those of the authors and do not necessarily represent those of their affiliated organizations, or those of the publisher, the editors and the reviewers. Any product that may be evaluated in this article, or claim that may be made by its manufacturer, is not guaranteed or endorsed by the publisher.

## Abbreviations

FHCM: Familial hypertrophic cardiomyopathy
ANP: Atrial natriuretic peptide
BNP: Brain natriuretic peptide
β-MHC: Beta-myosin heavy chain
PKC-α: Protein kinase C-alpha
NFAT: Nuclear factor of activated T cells
MEF2C: Myocyte enhancer factor 2C
Fgf12: Fibroblast growth factor 12
cAMP: Cyclic adenosine monophosphate
CREB: Cyclic adenosine monophosphate response-binding protein
RLC: Regulatory light chain
BSA: Bovine serum albumin
GAPDH: Glyceraldehyde-3-phosphate dehydrogenase
TBST: TBS+Tween
RNA-Seq: RNA Sequencing
DEGs: Differentially expressed genes
TGFβ: Transforming growth factor beta
TGFβR: Transforming growth factor beta receptor
Colq: Collagen-like tail subunit
Myh11: Heavy polypeptide 11
Ecm2: Extracellular matrix protein 2
Creb1: cAMP responsive element binding protein 1
FGFR4: Fibroblast growth factor receptor 4
PLC-γ: Phospholipase C gamma
MyHC: Myosin heavy chain.
