# Supplementary material for: Programmed Exercise Attenuates Familial Hypertrophic Cardiomyopathy in Transgenic E22K Mice via Inhibition of PKC-α/NFAT Pathway
Source: Front Cardiovasc Med. 2022 Feb 21;9:808163. doi: 10.3389/fcvm.2022.808163 (PMC8899095; doi:10.3389/fcvm.2022.808163)
Supplement: Supplementary file 1 [file Data_Sheet_1.docx]

**Caption for Supplemental Figures**

**Figure S1. E22K mutation elevates the protein expression of p-Smad2 and TGFβR**

Representative Western blot results demonstrated the upregulated expression of TGFβR and increased phosphorylation of Smad2 in R-Tg-E22K mouse hearts compared to those in R-Tg-WT and Non-Tg mouse hearts (A, C). Densitometric analysis of the protein levels of p-Smad2 and TGFβR in each group (B, D). N=3, *p<0.05; R-Tg-WT: wild-type transgenic mice in the rest group; R-Tg-E22K: transgenic E22K mice in the rest group; R-Non-Tg: non-transgenic wild-type mice in the rest group.

**Figure S2. E22K mutation upregulates the protein expression of PKC-α and NFAT**

Representative Western blot data showing the upregulated expression levels of PKC-α and NFAT in R-Tg-E22K mouse hearts compared to R-Tg-WT and R-Non-Tg mouse hearts (A, C). Densitometric analysis of the protein levels of PKC-α and NFAT in each group (B, D). N=3, *p<0.05; R-Tg-WT: wild-type transgenic mice in the rest group; R-Tg-E22K: transgenic E22K mice in the rest group; R-Non-Tg: non-transgenic wild-type mice in the rest group.

**Figure S3. Exercise changes the protein expression of PKC-α/NFAT**

Representative Western blot results show that there are no remarkable differences in the expression levels of PKC-α and NFAT between Tg-WT and exercise-trained E22K mice (A, C). Densitometric analysis of the protein levels of PKC-α and NFAT in each group (B, D). E-Tg-WT: wild-type transgenic mice in the exercise group; E-Tg-E22K: transgenic E22K mice in the exercise group; E-Non-Tg: non-transgenic wild-type in the exercise group.
