# Supplementary figures and images for: Programmed Exercise Attenuates Familial Hypertrophic Cardiomyopathy in Transgenic E22K Mice via Inhibition of PKC-α/NFAT Pathway
Source: Front Cardiovasc Med. 2022 Feb 21;9:808163. doi: 10.3389/fcvm.2022.808163 (PMC8899095; doi:10.3389/fcvm.2022.808163)

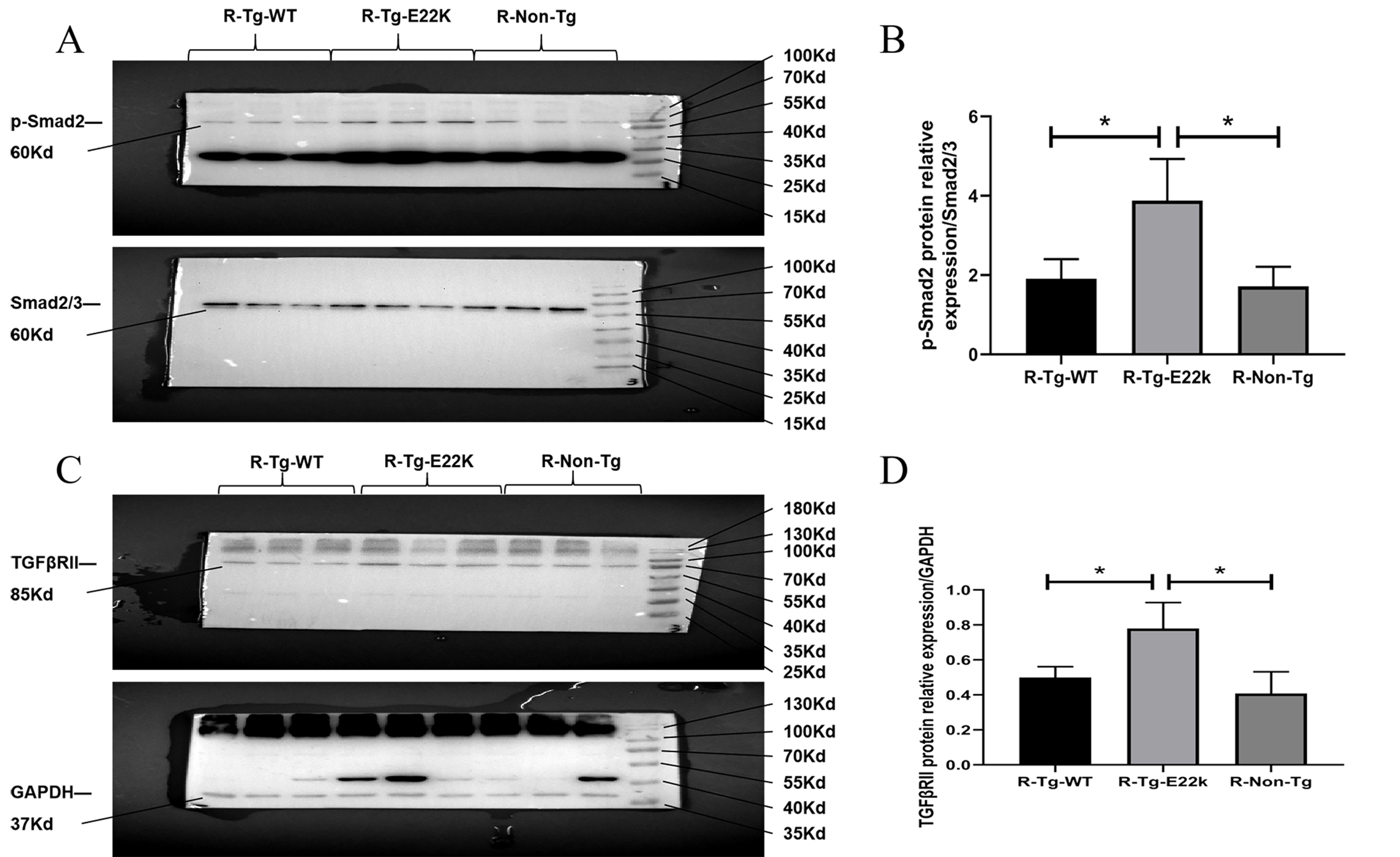

Supplement: Supplementary file 2 [file Image_1.JPEG]

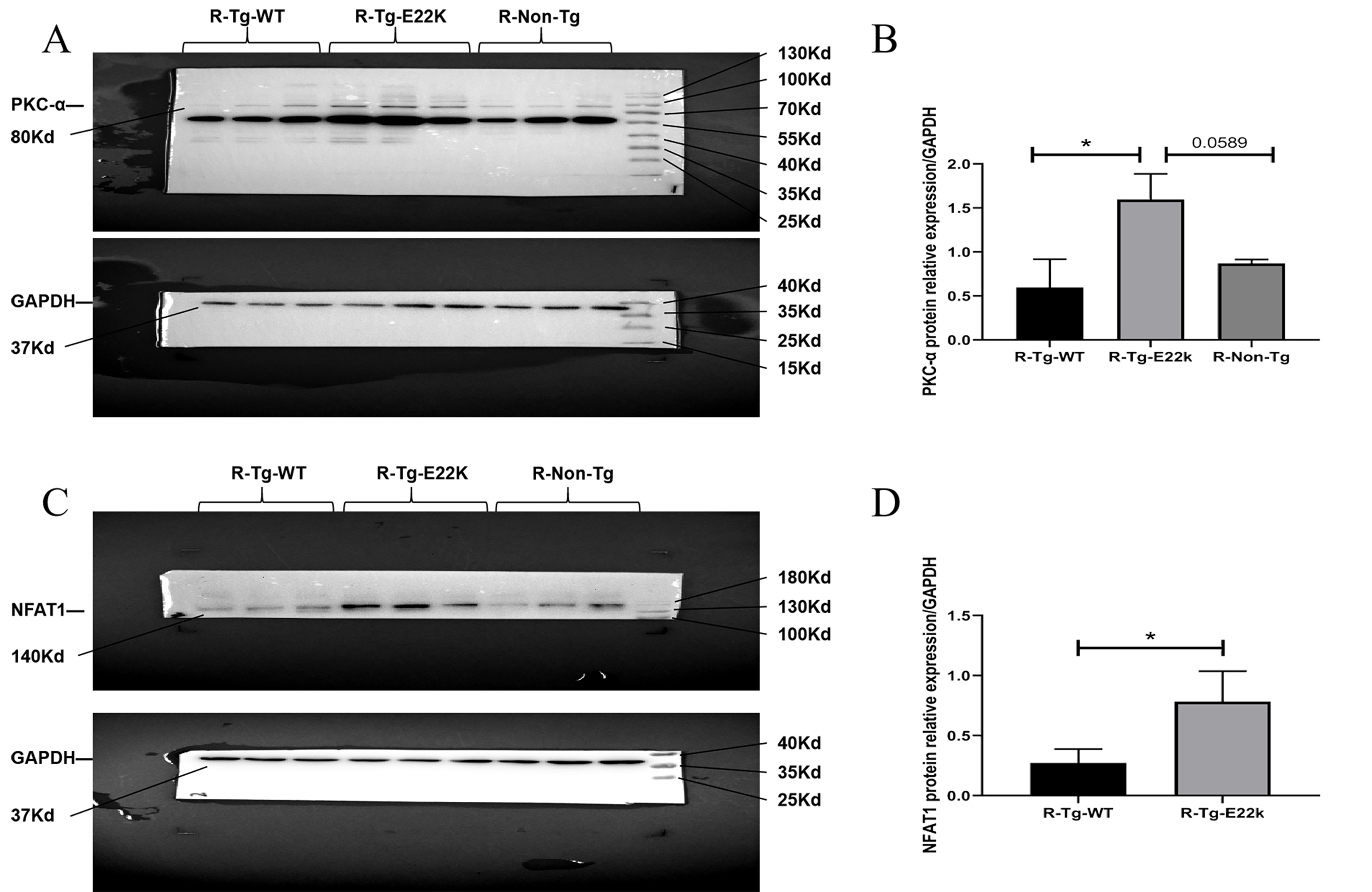

Supplement: Supplementary file 3 [file Image_2.JPEG]

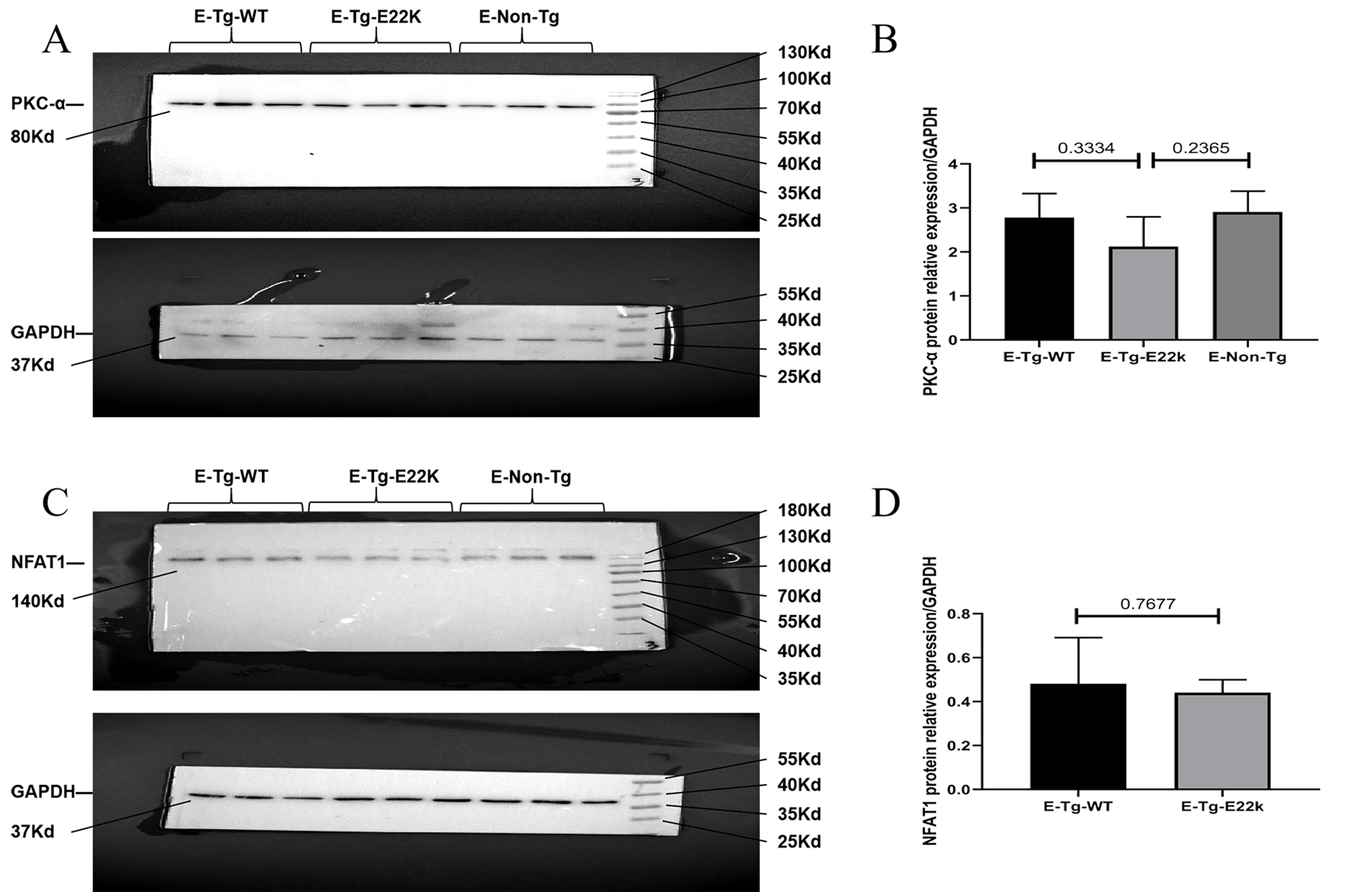

Supplement: Supplementary file 4 [file Image_3.JPEG]
